# Noradrenaline Increases mEPSC Frequency in Pyramidal Cells in Layer II of Rat Barrel Cortex via Calcium Release From Presynaptic Stores

**DOI:** 10.3389/fncel.2018.00213

**Published:** 2018-07-27

**Authors:** Julian M. C. Choy, Fransiscus A. Agahari, Li Li, Christian Stricker

**Affiliations:** ^1^Neuronal Network Laboratory, Eccles Institute of Neuroscience, The John Curtin School of Medical Research, Australian National University, Canberra, ACT, Australia; ^2^Division of Cerebral Circuitry, National Institute for Physiological Sciences, Okazaki, Japan; ^3^Zhejiang Provincial Key Laboratory of Anesthesiology, The Second Affiliated Hospital and Yuying Children’s Hospital, Wenzhou Medical University, Wenzhou, China; ^4^ANU Medical School, Australian National University, Canberra, ACT, Australia

**Keywords:** α_1_-adrenoceptor, presynaptic calcium stores, mEPSC, noradrenaline, neuromodulation

## Abstract

Somatosensory cortex is innervated by afferents originating from the *locus coeruleus* which typically release noradrenaline. We tested if activation of presynaptic α_1_-adrenoceptors (AR) coupled to a G_q_-mediated signaling cascade resulted in calcium (Ca^2+^) release from stores and thereby increased spontaneous transmitter release in rat barrel cortex. Adding 1–100 μM noradrenaline (NA) or 5 μM cirazoline (CO), a α_1_-AR specific agonist, to the standard artificial cerebrospinal fluid increased the frequency of miniature excitatory postsynaptic currents (mEPSC) by 64 ± 7% in 51% of pyramidal cells in layer II (responders) with no effect on the amplitude. In 42 responders, the mEPSC frequency during control was significantly smaller (39 ± 2 *vs.* 53 ± 4 Hz) and upon NA exposure, the input resistance (*R*_in_) decreased (9 ± 7%) compared to non-responders. Experiments using CO and the antagonist prazosin revealed that NA acted via binding to α_1_-ARs, which was further corroborated by simultaneously blocking β- and α_2_-ARs with propranolol and yohimbine, which did not prevent the increase in mEPSC frequency. To verify elements in the signaling cascade, both the phospholipase C inhibitor edelfosine and the membrane permeable IP_3_ receptor blocker 2-APB averted the increase in mEPSC frequency. Likewise, emptying Ca^2+^ stores with cyclopiazonic acid or the chelation of intracellular Ca^2+^ with BAPTA-AM prevented the frequency increase, suggesting that the frequency increase was caused by presynaptic store release. When group I metabotropic glutamate receptors were activated with DHPG, co-application of NA occluded a further frequency increase suggesting that the two receptor activations may not signal independently of each other. The increased mEPSC frequency in a subset of pyramidal cells results in enhanced synaptic noise, which, together with the reduction in *R*_in_, will affect computation in the network.

## Introduction

G_q_ protein-coupled receptor stimulation results in the activation of phospholipase Cβ (PLCβ) and the subsequent production of inositol-1,4,5-trisphosphate (IP_3_) and diacylglycerol (DAG; Berridge and Irvine, 1984). We have previously shown that IP_3_ production can result in an increased frequency of miniature excitatory postsynaptic currents (mEPSC; Simkus and Stricker, 2002b). As the rat somatosensory cortex receives a notable innervation by noradrenergic fibers from the *locus coeruleus* via a meshwork of axon terminals (Fuxe et al., 1968; Fritschy and Grzanna, 1990; Simpson et al., 2006; Agster et al., 2013), noradrenaline binding to α_1_-ARs may activate the same G_q_ cascade. In fact, specific binding of the α_1_-AR agonist 2-(β(4-hydroxyphenyl)-ethylamino-methyl)-tetralon in rat neocortex has been documented early on (Jones et al., 1985a,b). Furthermore, using *in situ* hybridization, α-ARs RNA transcripts have been found in neocortex of adult rats with slightly stronger staining for α_1_- than α_2_-AR (McCune et al., 1993). Moreover, Herold et al. (2005) and Jin et al. (2013) found an α_1_-AR-mediated increase in both excitability and spontaneous transmitter release in presympathetic and motor neurons in the rat and mouse spinal cord, respectively. However, in cortex the effects of α_1_-ARs activation on spontaneous transmitter release have been varied. While in hippocampal slice cultures, there was little effect (Scanziani et al., 1993), in prefrontal cortex a large increase in the frequency of spontaneous EPSCs in layer V pyramidal cells was reported by Marek and Aghajanian (1999). Given these discrepancies and the fact that the molecular mechanisms downstream of α_1_-ARs remained unclear, we re-evaluated the action of NA on spontaneous transmitter release in somatosensory cortex. We hypothesized that NA bound to presynaptic α_1_-ARs resulting in the activation presynaptic Ca^2+^ stores, which is seen as an increased mEPSC frequency.

Indeed, we found that in about half of all layer II pyramidal cells recorded from, upon exposure to 10 μM NA the mEPSC frequency increased with no concomitant change in amplitude. These cells were subsequently classified as responders. In the remaining cells (non-responders), no significant increases in mEPSC frequency were seen. In addition, in these responders, NA caused a significant reduction in input resistance while other postsynaptic parameters remained unchanged. We then show that the increase in mEPSC frequency was downstream of α_1_-ARs activation, as the α_1_-AR agonist cirazoline was able to fully reproduce the increase in mEPSC frequency, and the simultaneous blocking of α_2_- and β-ARs did not abolish the frequency increase. We then characterized each downstream signaling step in this cascade: blocking either PLCβ or IP_3_ receptors, emptying stores via SERCA pump block, or chelation of intracellular Ca^2+^ by BAPTA-AM all averted the frequency increase, suggesting that the frequency increase was caused by Ca^2+^ release from presynaptic stores. In addition, we provide evidence that after activation of group I metabotropic glutamate receptors by DHPG (Simkus and Stricker, 2002b), the co-application of NA did not result in a further increase in mEPSC frequency, suggesting that the downstream signaling of the two receptors may not be independent of each other.

## Materials and Methods

This study was carried out in accordance with the recommendations of Australian code for the care and use of animals for scientific purposes, 8*^th^* edition (2013), NHMRC. The protocol was approved by the Animal Ethics Committee of the Australian National University. Most methods are similar to the ones reported by Simkus and Stricker (2002a,b). In short, 300 μm thick slices of the posterior lateral subfield of the barrel cortex were prepared from 15 to 20 day-old Wistar rats using a vibratome (VT1200S; Leica). Slices were incubated at 34°C for at least 30 min and kept at room temperature until used for experiments.

Slices were visualized using IR-DIC (Stuart et al., 1993) at low magnification to find the barrels in layer IV of the medial posterior barrel subfield. In this area, pyramidal cells in the upper layer II were targeted for recording. All experiments were performed at 36 ± 1°C in artificial cerebrospinal fluid (ACSF) of the following composition (mM): NaCl, 125; KCl, 2.5; NaHCO_3_, 25; NaH_2_PO_4_, 1.25; CaCl_2_, 2.0; MgCl_2_, 1.0; glucose, 25.0, gassed with 95% O_2_ and 5% CO_2_, pH 7.4. Glass electrodes were pulled with a P97 (Sutter) using borosilicate glass (2/1 mm OD/ID; Hilgenberg) to a tip diameter of 3 – 4 μm (4 – 5 MΩ), which were filled with a solution of the following composition (mM): *K*-gluconate, 115; KCl, 20; HEPES, 10; phosphocreatine, 10; Mg-ATP, 4; Na-GTP, 0.3; biocytin, 5; pH 7.3; osmolarity, 303 mOsm. No correction was made for the junction potential (5.6 mV).

To isolate mEPSCs, 1 μM tetrodotoxin (TTX) was added to the superfusate together with the GABA_A_ receptor blocker gabazine (3 μM). Recordings were done in voltage-clamp at a holding potential of -70 mV using a Multiclamp 700A, the output of which was further amplified and filtered by a sample-and-hold amplifier (designed at JCSMR). Continuous recordings of mEPSCs for at least 5 min were filtered at 1 and acquired at 5 kHz using an ITC-18 (InstruTech) running custom-made routines in IGOR Pro 6.3. Before and after each recording sequence, the series resistance (*R*_s_) was checked. Cells were excluded from analysis if *R*_s_ was >20 MΩ, changed by >20% during the recording or the holding current (*I*_hold_) < -200 pA. Cells were included if positively identified histologically (see below) and/or had a pyramidal soma including an apical dendrite as judged from the DIC image, plus action potential properties and *R*_in_ consistent with those of pyramidal cells.

After recording, the slices were fixed in 4% paraformaldehyde for at least 2 h, subsequently washed in 0.1 M phosphate buffer and then incubated with avidin and biotin conjugated horseradish peroxidase (ABC Elite Kit, Vectastain) for 24–48 h in TRIS buffer containing 0.5% triton and later reacted with phenylenediamine and intensified with nickel and cobalt (Horikawa and Armstrong, 1988). After the addition of hydrogen peroxide (0.003%), the slices were left until the desired staining intensity was achieved. The tissues were subsequently mounted on slides in 0.9% Moviol^TM^. The slides with the cells were visualized and imaged on an Axioskop 2 MOT fitted with a digital CCD camera (Optronics). The criteria for determining whether a recovered cell was a pyramidal neuron were a pronounced apical dendrite studded with spines elongating into layer I, together with several basal dendrites emanating from the soma.

All chemicals were obtained from Sigma-Aldrich (Sydney, Australia) except for TTX (Latoxan, France), yohimbine hydrochloride (Research Biochemicals), (s)-3,5-dihydrophenylglycine (DHPG; Abcam, United Kingdom), cyclopiazonic acid (CPA), prazosin (PA), cirazoline (CO), 2-aminoethoxydiphenyl borate (2-APB), 1,2-bis-(2-aminophenoxy)ethane-*N*,*N*,*N*′,*N*′-tetraacetic acid (BAPTA-AM; Molecular Probes, United States), 2-hydropropyl-β-cyclodextrin (CD; Fluka, Switzerland), 1-octadecyl-2-O-methyl-glycero-3-phosphocholine (edelfosine, ES) and gabazine (Tocris).

Loading of BAPTA-AM (50 μM) was done according to Ouanounou et al. (1996). In short, BAPTA-AM was stabilized in the ACSF by 0.7 mM 2-hydropropyl-β-cyclodextrin and its intracellular accumulation was facilitated by the presence of 0.5 mM probenecid in the ACSF. The slice was loaded for at least 20 min, after which recordings resumed.

Ca^2+^ stores were emptied as described by Simkus and Stricker (2002b). In short, the SERCA pump inhibitor CPA (20 μM) was added to the ACSF for 20 min. Thereafter, while the cell was kept in current-clamp, a small drop of 3 M KCl (40 μl) was added directly to the recording chamber (5 ml) to cause a large depolarization to >5 mV lasting for several min. After ∼30 min, when the initial membrane potential was re-established, the recording was resumed in voltage-clamp. The idea behind this depolarization is to force Ca^2+^ release from stores which, when store uptake is blocked, efficiently empties them.

### Detection of mEPSCs

The technique for detecting mEPSCs was published in Simkus and Stricker (2002a). In short, the template-matching algorithm (Clements and Bekkers, 1997) implemented in AxoGraph 4.9 was used. Detection of mEPSCs by eye during the first 2 s of each recording was iteratively optimized to minimize the number of false positive and negative events. mEPSCs with amplitudes ≤2.5 times of the point-to-point noise standard deviation were discarded. This procedure uncovered typically 3,000 – 20,000 mEPSCs, for which the respective intervals, amplitudes, rise-times, and half-widths were determined. From the intervals, the instantaneous frequencies were calculated. A higher threshold value does not change the overall conclusions but increases the mean amplitude and reduces the frequency together with the significance levels. Note that the values of these instantaneous frequencies are larger than if the number of EPSCs is divided by the duration of the experiment (average frequency). This is because a Poisson process is dominated by short intervals, which results in a significantly larger average instantaneous frequency.

The average mEPSC time course was estimated after peak-aligning all mEPSCs. Because other mEPSCs commonly straddle the decay phase, the time courses were truncated typically after 50% of decay.

**FIGURE 1:**
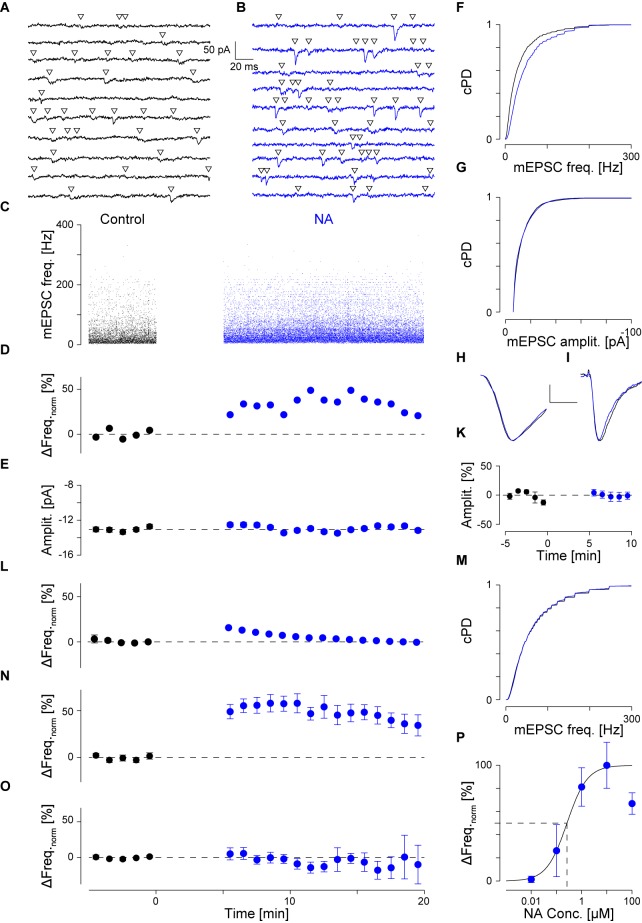
NA (10 μM) increases the mEPSC frequency without affecting the amplitude. **(A)** Continuous recording period (2 s) during control. Detected mEPSCs are indicated by a triangle. **(B)** Same as **(A)** but after addition of NA (blue). **(C)**. Individual instantaneous mEPSC frequencies in a single pyramidal cell shown for the control period (*t* < 0 min) and after addition of NA (*t* = 0 min). **(D)** Time course of minute averages (including respective errors) of the data in **(C)** after normalizing for the frequency during control (black) and after NA (blue). Dashed line indicates no change. **(E)** Time course of minute averages of mEPSC amplitudes. **(F)** Cumulative probability density functions of the instantaneous frequencies during control (black) and in NA (blue). **(G)** Same as in **(F)** but for the mEPSC amplitude. **(H)** Average mEPSC time course during control and in NA (scale bar 4 pA and 2 ms). **(I)** Average time course of iEPSCs during control and with 10 μM NA (scale bar 20 pA and 20 ms). **(K)** Normalized minute averages of all iEPSC amplitudes before and after NA. **(L)** Normalized minute averages of mEPSC frequency for a non-responder. **(M)** Same as for **(F)**, but the case in **(L)**. **(N,O)** Overall time courses of normalized minute averages of the mEPSC frequency in responders **(N)** and non-responders **(O)**, respectively. **(P)** Concentration-response relationship between normalized mEPSC frequency increase and NA concentration. The solid line is the best fit of a Hill equation to the data. Dashed lines indicate the *EC*_50_ value.

To determine *EC*_50_ for NA, data was gathered as follows. For concentrations ≤1 μM, the data was based on the full data set recorded; *i.e.*, both responders and non-responders are included. The maximal value at 10 μM used for subsequent normalization was derived from responders only. Note that at 100 μM there were only responders (**Figure 1L** and **Table 1**). The concentration-response relationship was estimated by fitting a Hill equation to the relative mEPSC frequency increase *vs.* chosen NA concentrations using the following relationship

Relative⁢ EPSC⁢ frequency=11+(EC50[NA])c,

**Table 1:** Overview of outcomes under different experimental conditions.

Condition	Subset	mEPSC Frequency [Hz]			*p*_pt_/*p*_B_
			
		Control	Agonist	Δ [%]	
0.01 μM NA	Responders (0)	–	–	–	–
	Non-responders (3)	52 ± 10	53 ± 11	1 ± 2	0.28
0.1 μM NA	Responders (2)	30 ± 1	48 ± 11	61 ± 32	0.16
	Non-responders (5)	45 ± 11	46 ± 14	-1 ± 7	0.36
1 μM NA	Responders (8)	43 ± 3	64 ± 4	52 ± 11	*0.0006*
	Non-responders (0)	–	–	–	–
10 μM NA	Responders (12)	39 ± 3	61 ± 4	64 ± 13	*3⋅10^-5^*
	Non-responders (25)	59 ± 5	58 ± 5	-1 ± 4	0.37
100 μM NA	Responders (4)	32 ± 3	47 ± 6	43 ± 6	*0.008*
	Non-responders (0)	–	–	–	–
10 μM NA (with YO/PO)	Responders (8)	37 ± 6	60 ± 5	81 ± 23	*0.0001*


	Non-responders (0)	–	–	–	–
5 μM CO	Responders (8)	43 ± 5	69 ± 5	72 ± 21	*0.001*
	Non-responders (7)	37 ± 4	37 ± 3	3 ± 8	0.50


**The number of respective recordings is given in brackets. Because the individual entries were calculated based on the average changes for individual cells, the 5^*th*^ column is not precisely the difference between the preceding two columns. Significant values are italicized. p_*pt*_ indicates paired t-test and p_*B*_ post hoc t-test with Bonferroni correction.**

with *EC*_50_ the effective concentration at half the relative frequency increase and *c* the cooperativity factor. The fit and the error estimates of the parameters were determined using the built-in optimization in IGOR Pro. This implementation iteratively minimizes the χ^2^ values based on a Levenberg–Marquardt algorithm (Press et al., 1992). The errors of the parameters were estimated according to a linear approximation of the underlying distribution (see chapter 15 in Press et al., 1992).

### Iontophoretically Evoked EPSCs (iEPSCs)

To check for postsynaptic effects of some of the pharmacological compounds used, iontophoresis of AMPA onto apical dendrites was done with fine microelectrodes (100–200 MΩ when backfilled with 10 mM AMPA) pulled from 1.2 mm O.D. borosilicate glass (Clark Electromedical). An Axoclamp 2B (Molecular Devices) with a x1 headstage was used in combination with an isolated stimulator (DS-2, Digitimer Ltd.), which provided command voltages of up to 100 V. iEPSCs were evoked using current stimuli of ∼-600 nA at a frequency of 0.2 Hz. In between stimuli, AMPA leakage from the electrode tip was minimized by a constant bias current of 5 nA. A few negative control experiments for some of the drugs used in this study have been published before (Simkus and Stricker, 2002a).

### Statistical Analyses

To compare mEPSC characteristics during control and after NA, cumulative probability density functions (cPDFs) were formed and compared using the Kolmogorov–Smirnov (KS) statistic (*p*_KS_). Since these cPDFs were based on large samples, the significance level for this statistic was usually taken at <10^-6^. If the difference in frequencies was significant, the cell was classified as a responder or non-responder otherwise.

If there was no statistical difference, the data from different exposures were pooled and analyzed as follows. If there were only two groups to consider, a Student *t*- or paired *t*-test (*p*_t_ or *p*_pt_) was appropriate. Multiple sets of experiments were compared using a one-way ANOVA (*p*_O_). If multiple measures in the same set of experiments were compared, ANOVA of repeated measures (*p*_ANOVA_) was performed. If *p*_ANOVA_ < 0.05, the differences between each of the different measures was assessed using a *post hoc t*-test with Bonferroni correction (*p*_B_). In this case, for comparing three different measures, the significance level becomes 0.017. Otherwise, statistical significance was judged at ≤0.05. Error bars associated with parameter values indicate mean ± SEM. Note that the mean values and the changes associated presented throughout are based on the respective samples rather than the estimation between the respective means.

## Results

We recorded pharmacologically isolated mEPSCs in identified pyramidal cells of layer II in rat barrel cortex while blocking GABAergic currents with 3 μM gabazine and sodium channels with 1 μM tetrodotoxin (TTX). The average membrane potential of these cells was -76.4 ± 3.9 mV (*n* = 189) and their input resistance 120 ± 5 MΩ (ranging from 28 to 577 MΩ). The average series resistance (*R*_s_) was 10.5 ± 0.2 MΩ and after filtering at 1 kHz, the standard deviation of the whole-cell noise (σ*_n_*) during periods without obvious mEPSCs was 2.61 ± 0.03 pA (range 1.3–3.9 pA).

### Noradrenaline Increases mEPSC Frequency but Not Amplitude

An example of a typical recording is shown in **Figure 1**. Two seconds of continuous recording are shown during the control period (**Figure 1A**, black throughout) and after addition of 10 μM NA (**Figure 1B**, blue throughout). Detected mEPSCs are marked by an open triangle. During control, a total of 4,777 mEPSCs were detected, the individual intervals of which were determined to calculate the instantaneous frequencies in **Figure 1C** (*t* < 0 min). The average instantaneous frequency was 39 ± 1 Hz and the amplitude -13.0 ± 0.1 pA. After the addition of 10 μM NA at *t* = 0 min (**Figure 1C**) and the resumption of recording after a gap of 5 min, 22,114 mEPSCs were detected during the subsequent 15 min. The respective instantaneous frequencies are presented in **Figure 1C** for *t* ≥ 5 min. The average instantaneous frequency during this period was 53 ± 1 Hz, which corresponds to an increase by 34 ± 3%. After normalizing for the average value during control, the minute averages of the frequencies are shown for this example in **Figure 1D** during control (black) and after the addition of NA (blue). The frequency increase was maintained throughout the period of exposure to NA. The cumulative probability density functions (cPDF) of the respective frequencies during control and after NA are given in **Figure 1F** indicating a very significant increase in frequency (*p*_KS_ < 10^-6^).

An increase in frequency could have arisen if the mEPSC amplitude became larger in NA. We therefore checked if the mEPSC amplitude increased. In **Figure 1E**, the time course of the minute averages for the respective mEPSC amplitudes is shown, indicating that throughout the recording, minimal deviations from the control value (-13.0 ± 0.1 pA) were observed. The respective cPDFs for the amplitudes are given in **Figure 1G**, indicating that no significant increase in mEPSC amplitude between the two periods was observed. To cross-check that no significant changes in mEPSC kinetics and amplitude occurred, all mEPSC time courses were peak-aligned and averaged before (black) and after NA (blue; **Figure 1H**). There were no significant changes in mEPSC kinetics or amplitude supporting the idea that the frequency increase is due to a change in the rate of vesicle release.

To further corroborate that the mEPSC amplitude did not change in NA and a postsynaptic effect was unlikely, we performed experiments in which, from a high resistance electrode (>80 MΩ) containing 10 mM AMPA, iontophoretic EPSCs (iEPSC) were evoked onto the apical dendrite without and in the presence of 10 μM NA. An example of such an average iEPSC time course is shown in **Figure 1I**, where during the control period (black) the amplitude was -75.0 ± 2.1 and in the presence of NA -74.5 ± 2.8 pA (blue). In a set of 12 such experiments, the average iEPSC amplitude during control and in the presence of NA was -55.9 ± 4.7 and -55.5 ± 4.4 pA, respectively, which was insignificantly altered. The relative change in iEPSC amplitude is presented in minute averages in **Figure 1K** showing that there were minimal changes after exposure to NA. Similar outcomes were seen for the respective rise times and half-widths, indicating that NA had no or a minimal effect on both the iEPSC amplitude and kinetics. Therefore, the frequency increase by NA was unlikely caused by an increase in mEPSC amplitude or by unsilencing of postsynaptic AMPA receptor patches.

To also rule out that the increase in mEPSC frequency was an artifact incurred by the solution change or was due to random variations in the rate of mEPSCs in the slice, we did recordings over 40 min including a switch to the same ACSF but from a different container (like when NA was added). In this set (*n* = 7; data not shown), the average mEPSC frequency and amplitude were 39 ± 4 Hz and -14.0 ± 0.8 pA, respectively; *i.e.*, not different to that observed during control conditions (*p*_pt_ = 0.77 and 0.19, respectively). No significant changes in either the mEPSC frequency or amplitude were observed throughout the recordings in this set. This strengthens the idea that the increase in mEPSC frequency is caused by an increased rate of mEPSCs.

However, we noticed that this increase in mEPSC frequency was not observed in all pyramidal cells recorded from. Such an outcome is illustrated in **Figure 1L**. In this cell, the average mEPSC frequency during control was 59 ± 2 Hz. After the exposure to NA, there were no changes to either the normalized frequency or the cPDFs (*p*_KS_ = 0.004; **Figure 1M**); *i.e.*, the frequency was maintained throughout. There were also no changes to the mEPSC amplitudes as reported above. We subsequently referred to these cells as non-responders and those with a significant increase in mEPSC frequency as responders. The time courses of the relative change in frequency for all responders (*n* = 42) is illustrated in **Figure 1N** and for non-responders (*n* = 40) in **Figure 1O**. More details are provided below.

We checked if there were significant differences in the cellular properties between these two groups. During control, *R*_in_ for responders was 122 ± 8 and for non-responders 126 ± 12 MΩ (*p*_t_ = 0.80); *i.e.*, there was no difference between the two groups. However, after agonist exposure, *R*_in_ for responders dropped by 9 ± 7% to 103 ± 7 MΩ (*p*_pt_ = 0.01) but remained unchanged for non-responders (109 ± 10 MΩ; *p*_pt_ = 0.06). This suggests that NA opened a postsynaptic conductance in responders, whereas an insignificant change was observed in non-responders. These differences were unlikely due to variation in *R*_s_ as the values for responders and non-responders were 11.2 ± 0.5 and 11.1 ± 0.6 before and 10.9 ± 0.5 and 11.9 ± 0.6 MΩ after NA, respectively.

Comparing the two groups, we found that in responders the mEPSC frequency during control was significantly smaller than in non-responders (39 ± 2 *vs*. 53 ± 4 Hz; *p*_t_ < 10^-3^). In responders, though, the frequency after NA exposure was larger than the frequency during control in non-responders (61 ± 2 *vs*. 53 ± 4 Hz; *p*_t_ = 0.04), suggesting that NA increased the frequency in responders beyond that in non-responders. In regard to the mEPSC amplitude, there were no differences between the two groups before and after agonist exposure (-13.2 ± 0.5 *vs*. -12.8 ± 0.5 pA for before, and -12.9 ± 0.4 *vs*. -12.3 ± 0.4 pA for after NA application; *p*_t_ > 0.40).

Next, we determined the concentration-response relationship between the normalized relative frequency increase in responders and the NA concentration. The relevant data are summarized in **Table 1** and presented **Figure 1P** as a semi-logarithmic plot. A Hill equation was fitted to these data with an *EC*_50_ of 0.26 ± 0.19 μM and a cooperativity of 1.1 ± 0.6. The plot suggests that 10 μM NA is a saturating concentration and that, on average, one NA molecule binds to each AR.

### Involvement of α_1_-ARs

**FIGURE 2:**
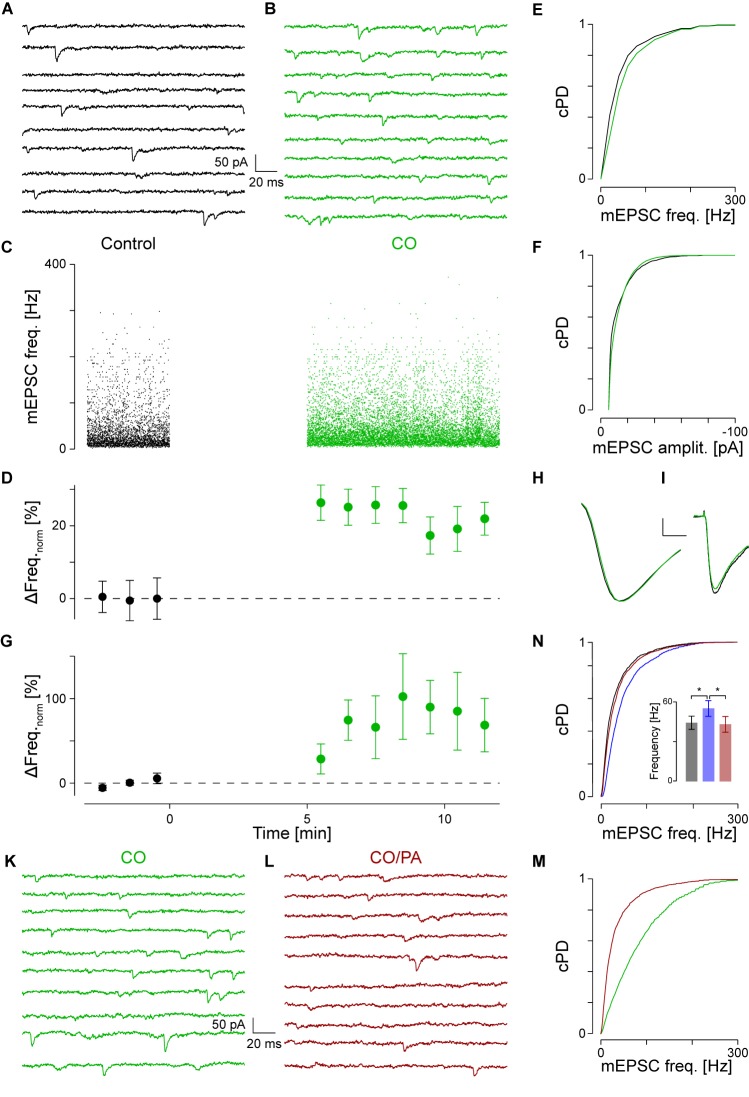
NA activates α_1_-AR. **(A)** Recording period of 2 s before and in **(B)** after application of CO (green). **(C)** Individual instantaneous mEPSC frequencies shown for the control period (*t* < 0) and after addition of CO (*t* > 0). **(D)** Time course of minute averages after normalizing for the frequency during control (black) and after CO (green). **(E)** cPDFs of the instantaneous mEPSC frequencies during control and after addition of CO. **(F)** Same as in **(E)** but for the mEPSC amplitude. **(G)** Average time course of responders during control and after exposure to CO. **(H)** Average mEPSC time course during control and after CO (scale bar 2 pA and 1 ms). **(I)** Average iEPSC time course during control and with CO (scale bar 10 pA and 25 ms). **(K)** Recording period in the presence of CO. **(L)** Recording period after the displacement of CO by prazosin (brown). **(M)** cPDFs of the instantaneous mEPSC frequencies in the presence of CO and after displacement with prazosin. **(N)** cPDFs for control (black), after NA (blue) and after displacement with prazosin (brown). Inset: Average mEPSC frequencies during control (black), after NA (blue) and in the presence of PA (brown). ^∗^*p*_B_ < 0.01.

To identify the type of AR to which NA binds, 5 μM cirazoline (CO) was used, which is a competitive partial agonist at α_1_- and an antagonist at α_2_-ARs (Ruffolo and Waddell, 1982). The results of a single experiment are shown in **Figures 2A,B**, where two representative periods are shown before (**Figure 2A**) and after addition of CO (**Figure 2B**). In this example, CO increased the instantaneous mEPSC frequency by 24 ± 4% from 45 ± 1 to 55 ± 1 Hz (*p*_KS_ < 10^-6^, **Figures 2C,E**). The time course of the minute averages of the normalized mEPSC frequency for this cell is presented in **Figure 2D**. Again, the mEPSC amplitude did not change (**Figure 2F**). In a total set of 15 such recordings, the mEPSC frequency increased by 40 ± 15% from 40 ± 3 to 54 ± 5 Hz (*p*_pt_ = 0.01). However, according to the grouping above, there were eight responders in this set. For these, CO increased the mEPSC frequency by 72 ± 21% (43 ± 5 *vs*. 69 ± 5 Hz; *p*_pt_ = 0.001; **Figure 2G**). This frequency increase was not different to the one observed with NA (*p*_t_ = 0.44). In addition, there were insignificant changes in mEPSC amplitude (**Figure 2H**) and kinetics as corroborated with iontophoresis (**Figure 2I**).

To exclude an alternative action of NA via α_2_- and β-ARs, these receptors were blocked with yohimbine (YO, 1 μM) and propranolol (PO, 1 μM), respectively. In this set of experiments (*n* = 8), the average frequency during control was 37 ± 6 Hz, but after addition of NA increased by 81 ± 23% to 60 ± 5 Hz (*p*_pt_ < 10^-4^; data not shown). This increase was not different to the one observed without any receptor blockers (NA *vs*. CO *vs*. PO/YO; *p*_O_ = 0.66). Again, no significant changes were observed for mEPSC amplitudes (-14.9 ± 1.0 *vs*. -14.1 ± 1.3 pA, respectively; *p*_pt_ = 0.41). This data indicates that neither α_2_- nor β-ARs play a significant role in modulating miniature transmitter release.

To provide even more evidence that the frequency increase was caused by specific binding to α_1_-AR, displacement experiments of CO with the competitive antagonist prazosin (5 μM, PA; Hornung et al., 1979; Miach et al., 1980) were performed. Results of such an experiment are shown in **Figures 2K–M**. In the presence of CO, the mEPSC frequency was 55 ± 2 Hz (**Figure 2K**). After addition of PA and in the presence of CO, the mEPSC frequency dropped back by 33 ± 3% to 37 ± 1 Hz (*p*_KS_ < 10^-6^, **Figures 2L,M**). There were no significant changes in mEPSC amplitude or kinetics after addition of PA. In four such experiments, the average normalized frequency in responders decreased by 49 ± 7% (*p*_ANOVA_ = 0.003; *p*_B_ = 0.005) with no changes observed for amplitude or time course. This frequency value was not different to the control condition (*p*_B_ = 0.07), indicating that at this concentration, PA fully displaced CO from the receptor.

We cross-checked if PA also displaced NA from α_1_-ARs. The results of a single experiment are shown in **Figure 2N**. In this example, NA significantly increased mEPSC frequency by 49 ± 2% from 37 ± 1 to 55 ± 1 Hz, with a significant drop by 24 ± 1% to 42 ± 2 Hz after the displacement with PA. Again, no significant changes in mEPSC amplitude were observed. In a set of five experiments, NA increased the mEPSC frequency by 63 ± 16% (**Figure 2N** inset; *p*_ANOVA_ = 0.012; *p*_B_ = 0.005), but after addition of PA decreased by 23 ± 6%. This frequency was again not different to that in control condition (*p*_B_ = 0.14). This data is consistent with competitive displacement of NA by PA and strengthens the idea that NA caused an increase in spontaneous transmitter release via activation of α_1_-ARs.

As PA was dissolved in 8 μM DMSO, control experiments assessing the effect of PA on release showed that neither the mEPSC frequency (52 ± 141 *vs*. 53 ± 13 Hz; *n* = 5), amplitude (-13.7 ± 0.81 *vs*. -13.6 ± 0.8 pA), nor kinetics changed, indicating that at the concentration used, DMSO had minimal effects on mEPSCs.

As there were no statistical differences between experiments using NA or CO (*p*_t_ = 0.44), we pooled the data to gain accuracy and more statistical power. For responders (*n* = 42; see **Table 1**), the average increase in mEPSC frequency was 64 ± 7% from 39 ± 2 to 61 ± 2 Hz (**Figure 1N**; *p*_pt_ < 10^-14^), without any change in amplitude; *i.e.*, -13.2 ± 0.5 and -12.9 ± 0.4 pA, respectively (**Figure 1O**; *p*_pt_ = 0.23). However, for non-responders (*n* = 40), there was no increase (0 ± 3%; *p*_pt_ = 0.45), which was not different from that during control. Likewise, there was also no significant change in mEPSC amplitude (data not shown). Note, even when the data from responders and non-responders was pooled, the mEPSC frequency remained significantly increased (33 ± 5%; *p*_pt_ = 3⋅10^-9^), suggesting that the increase is robust to the “dilution” by non-responders.

### Mechanism of mEPSC Frequency Increase

**FIGURE 3:**
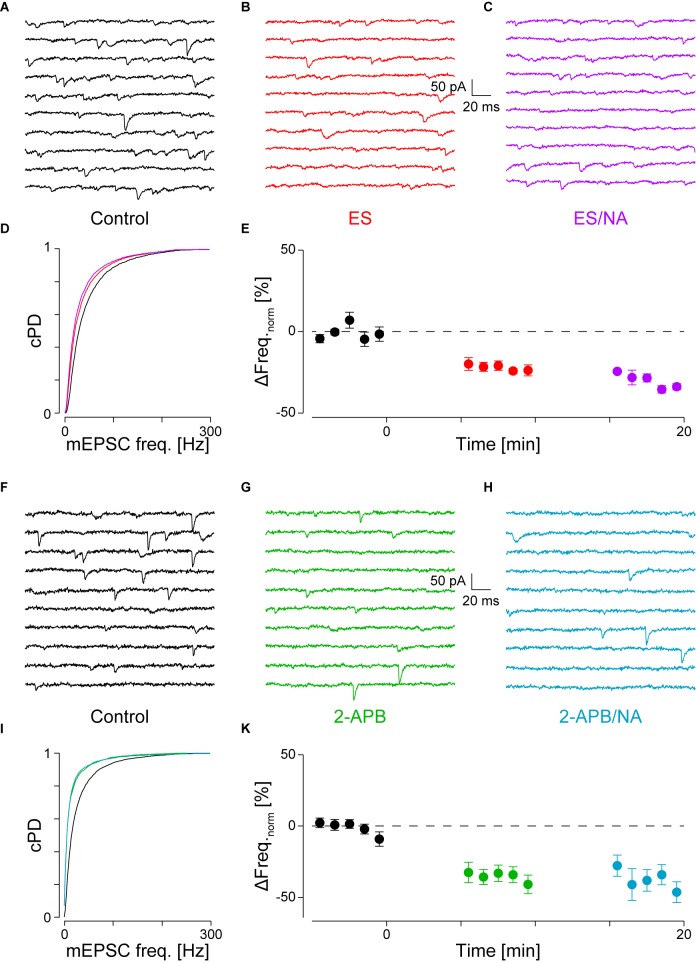
With PLCβ or IP_3_R blocked, there is no NA-mediated frequency increase. **(A)** Recording period before, **(B)** after addition of ES (red) and **(C)** when NA was co-applied (purple). **(D)** cPDFs of the instantaneous mEPSC frequencies before, after the addition of ES and when NA was co-applied. Same color code as above. **(E)** Time course of minute averages of the normalized mEPSCs frequencies before (*t* < 0 min), after the addition of ES (*t* > 0 min) and after the co-application of NA with ES (*t* > 10 min; *n* = 4). **(F–K)** Same as in **(A–E)**, but for the case where IP_3_R were blocked with 2-APB (green) and then stimulated with NA (turkish).

**FIGURE 4:**
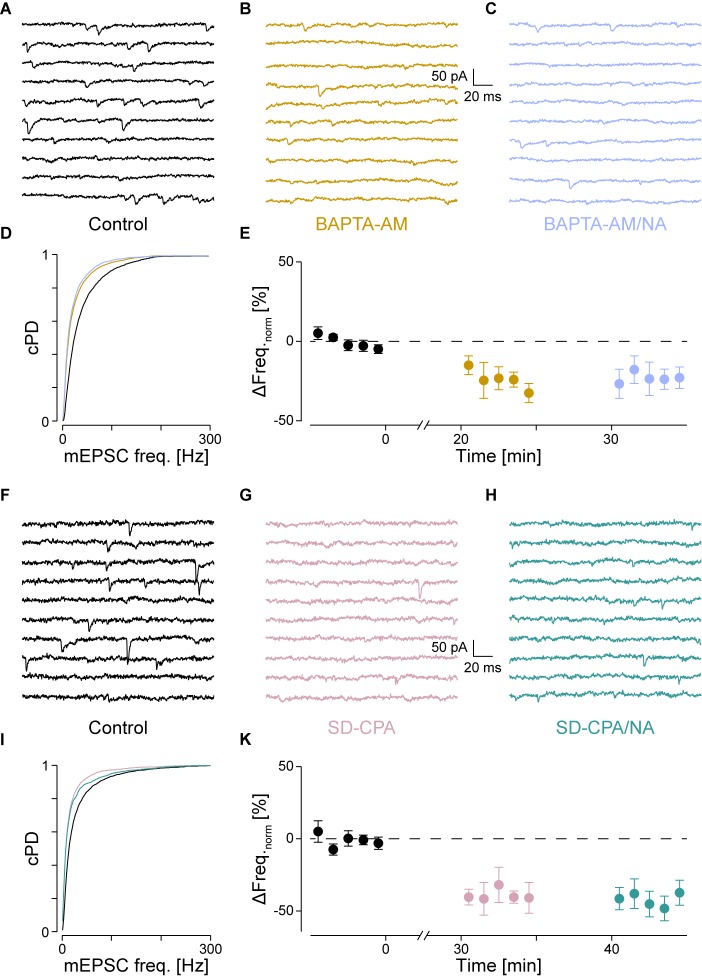
With intracellular Ca^2+^ chelated or stores depleted, there is no NA-mediated frequency increase. **(A)** Recording period before (black), **(B)** after Ca^2+^ chelation (dijon) and **(C)** when NA was then co-applied (cornflower). **(D)** cPDFs of the instantaneous mEPSC frequencies before, after BAPTA-AM and when NA was then co-applied. **(E)** Time course of the minute averages of the normalized mEPSCs frequencies before, after BAPTA-AM and co-application of NA (*n* = 4). **(F)** Recording period before, **(G)** after store depletion with CPA and K^+^ depolarization (SD-CPA; taffy), **(H)** and when NA was then co-applied (steel). **(I)** Respective cPDFs of the instantaneous mEPSC frequencies before, after store depletion and when NA was then co-applied. **(K)** Time course of the minute averages of the normalized mEPSCs frequencies before (black; *t* < 0 min), after store depletion (taffy; *t* > 0 min) and subsequent co-application of NA (steel; *t* > 35 min; *n* = 5).

**FIGURE 5:**
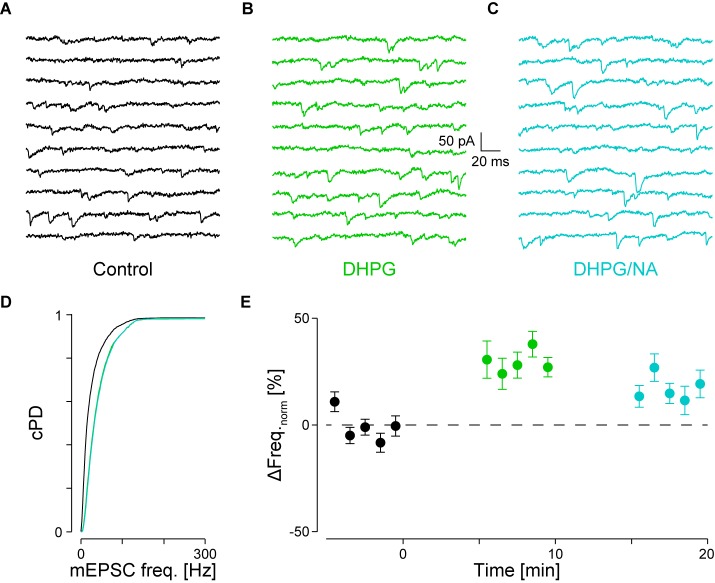
Occlusion of NA-mediated frequency increase when DHPG is present. **(A)** Recording periods before (black), **(B)** after addition of DHPG (mint) and **(C)** subsequent co-application of NA (maya). **(D)** Respective cPDFs of the instantaneous mEPSC frequencies before, after application of DHPG and **(C)** after co-application of NA. **(E)** Time course of the minute averages of the normalized mEPSCs frequencies before (*t* < 0 min), after addition of DHPG (*t* > 0 min) and subsequent co-application of NA (*t* > 10 min; *n* = 9).

G_q_-mediated signaling downstream of α_1_-AR stimulation involves activation of PLCβ, which results in the hydrolysis of phosphatidylinositol-4,5-bisphosphate (PIP_2_) into DAG and IP_3_. The latter could bind to IP_3_R on stores to cause Ca^2+^ release, which, in turn, may result in the observed increase in mEPSC frequency. We subsequently verified this “classic” signaling cascade. In the subsequent **Figures 3**–**5**, we only show averages of neurons that showed a decrease in frequency upon exposure to the respective blockers.

### Activation of Phospholipase Cβ

If α_1_-AR activation resulted in the hydrolysis of PIP_2_ to produce IP_3_ and DAG, then blockade of PLCβ in the presence of NA should prevent the increase in mEPSC frequency. For this purpose, we used the PLC inhibitor edelfosine (ES, 30 μM; Powis et al., 1992). Such an experiment is shown in **Figures 3A–D**, where in A, a short period during control, in B after the addition of ES and in C, in the presence of ES, but with NA added. Compared to control, the addition of ES to the superfusate significantly decreased the mEPSC frequency by 20 ± 3% (from 48 ± 1 to 39 ± 1 Hz, *p*_KS_ < 10^-6^; **Figure 3D**), but not amplitude. When NA was added in the presence of ES (C), the mEPSC frequency increase was blocked (**Figure 3D**). There was also no change in the amplitude.

The time course of the minute averages of the normalized average mEPSC frequency of four such experiments is shown in **Figure 3E** and **Table 2** for a period of 5 min each. Note that in this set of experiments, there are three different outcomes, which in the first instance were analyzed using ANOVA of repeated measures followed by *post hoc t*-tests (Bonferroni). ES caused the frequency to decrease by 22 ± 3% from 45 ± 2 to 35 ± 2 Hz (*p*_ANOVA_ < 10^-5^, *p*_B_ < 0.001). Subsequent addition of NA did not increase the mEPSC frequency (32 ± 2 Hz, *p*_B_ = 0.023) with no effect on the amplitude (-14.2 ± 0.8 *vs*. -14.3 ± 1.1 *vs*. -13.3 ± 0.8 pA, respectively; *p*_ANOVA_ = 0.14). This data shows that there is tonic PLCβ activity in these presynaptic nerve terminals and that, when PLCβ was blocked, there was no increase in mEPSC frequency; *i.e.*, the action of NA was averted.

**Table 2:** mEPSC frequency changes when downstream signaling was blocked by edelfosine (ES), 2-APB, BAPTA or after depletion of stores (DS).

mEPSC frequency [Hz]	ES (4)	2-APB (10)	BAPTA (4)	DS (5)	*p*_O_
Control	45 ± 2	49 ± 5	40 ± 3	41 ± 9	
Blocker	35 ± 2	33 ± 4	30 ± 3	26 ± 8	
*p*_B_	*0.0006*	*0.0001*	0.018	*0.002*	
Δ [%]	-22 ± 3	-34 ± 5	-24 ± 6	-40 ± 6	*0.18*


**The number of respective recordings given in brackets. Significant values are italicized. p_*O*_ indicates one-way ANOVA and p_*B*_ post hoc t-test with Bonferroni correction. Δ represents the change.**

### Activation of IP_3_ Receptors

If PLCβ activation resulted in the production of IP_3_ and subsequent opening of IP_3_R to cause Ca^2+^ release from stores, the block of these receptors should also prevent the NA-mediated increase in mEPSC frequency. We used the membrane-permeable IP_3_ receptor blocker 2-aminoethoxydiphenyl borate (2-APB, 16 μM), which at this concentration is quite specific (Simkus and Stricker, 2002b; Hirst et al., 2008). The results of this type of experiment are shown in **Figures 3F–I**. In this example, during the control period, the mEPSC frequency was 33 ± 1 Hz (**Figure 3F**). After addition of 2-APB, the mEPSC frequency significantly decreased by 46 ± 3% to 18 ± 1 Hz (**Figure 3G**) without significantly altering the amplitude or time course. When in the presence of 2-APB NA was then added, no subsequent changes in the frequency (**Figures 3H,I**) or amplitude were observed.

In eight such experiments, the mEPSC frequency initially dropped by 34 ± 5% from 49 ± 5 to 33 ± 4 Hz (*p*_pt_ = 10^-4^; **Figure 3K** and **Table 2**). Subsequent exposure to NA did not affect the frequency (*n* = 4, *p*_ANOVA_ = 0.013, *p*_B_ = 0.17). In both cases, there was no change to the amplitude or time course. We checked if the decrease in mEPSC frequency with 2-APB was comparable to that when PLCβ was blocked and found that they were indistinguishable (22 ± 3 *vs*. 34 ± 5%; *n* = 4 and 10, respectively; *p*_O_ = 0.18 for four groups; see below). This data shows that when IP_3_ receptors are blocked, the action of NA is also averted. This finding suggests that the tonic IP_3_ production is largely caused by constitutive PLCβ activity.

### Ca^2+^ Release From Presynaptic Stores

If IP_3_ binding caused Ca^2+^ release from stores, then either chelating Ca^2+^ with BAPTA or depleting stores should prevent the increase in mEPSC frequency. To test the first possibility, we loaded the slices with the membrane permeable Ca^2+^ chelator BAPTA-AM (50 μM) as described in the Methods. As we have previously shown that block of voltage-dependent Ca^2+^ channels did not affect the frequency increase (Simkus and Stricker, 2002b), the action of BAPTA is largely restricted to intracellular Ca^2+^ release. The results for a single cell are shown in **Figure 4A** for the control condition, in **Figure 4B** after loading with BAPTA, and in **Figure 4C** after subsequent exposure to NA. The loading of BAPTA for 20 min significantly decreased the mEPSC frequency by 38 ± 3% (from 42 ± 1 to 26 ± 1 Hz; *p*_KS_ < 10^-6^), without significantly altering the amplitude or time course. After NA was then added, no increase of either the mEPSC frequency (24 ± 1 Hz) or amplitude was observed.

In a set of four such experiments, the mEPSC frequency decreased by 24 ± 6% from 40 ± 3 to 30 ± 3 Hz (*p*_ANV OA_ = 0.017; *p*_B_ = 0.018; **Figure 4E** and **Table 2**). When NA was then added, neither the mEPSC frequency (30 ± 3 Hz, *p*_B_ = 0.44) nor amplitude changed (-10.0 ± 0.8 *vs*. -9.6 ± 0.7 pA; *p*_B_ = 0.07). Comparing this decrease with that observed when PLCβ or when IP_3_R were blocked (see above) revealed no difference (*p*_O_ > 0.18). Given that the frequency increase remained, this set of experiments indicates that tonic Ca^2+^ release from presynaptic stores causes a mEPSC frequency increase of ∼20–30%, which was blocked when BAPTA was loaded into the nerve terminals.

To test the second possibility, we depleted intracellular Ca^2+^ stores as described in the Methods. In **Figures 4F–I**, the results of an individual experiment are shown. Store depletion in the presence of CPA significantly decreased the mEPSC frequency by 43 ± 3% (from 33 ± 1 to 19 ± 1 Hz, *p*_KS_ < 10^-6^), without any changes to the mEPSC amplitude or time course. Subsequent exposure to NA in the presence of CPA (**Figure 4H**) failed to alter either the frequency or amplitude. In five such experiments, the average frequency decreased by 40 ± 6% from 41 ± 9 to 26 ± 8 Hz (*p*_ANOVA_ < 0.001; *p*_B_ = 0.0017; **Figure 4K** and **Table 2**), while subsequent application of NA was unable to increase the mEPSC frequency (26 ± 9 Hz; *p*_B_ = 0.48) or amplitude. This set of experiments further supports the idea that when stores are depleted of Ca^2+^, α_1_-ARs signaling is prevented.

A set of control experiments was made to demonstrate that the K^+^ depolarization did not affect subsequent miniature release. In 19 such experiments, the mEPSC frequencies and amplitudes during control *vs*. after a K^+^ depolarization were 43 ± 3 *vs*. 42 ± 4 Hz (*p*_pt_ = 0.73) and -14.7 ± 0.8 *vs*. -15.6 ± 1.1 pA (*p*_pt_ = 0.83), respectively, indicating that this depolarization neither appreciably affected the frequency nor amplitude of the mEPSCs. Without such a depolarization, there was no decrease in mEPSC frequency (data not shown), consistent with what was reported earlier (Simkus and Stricker, 2002b).

After receptor activation, some G proteins dissociate in a voltage-dependent manner (Ben-Chaim et al., 2003). Accordingly, the K^+^ depolarization may have affected subsequent downstream signaling. To assess this possibility, we checked if after K^+^ depolarization, the mEPSC frequency still increased after NA. In a set of seven out of nine experiments, subsequent addition of NA still increased the mEPSC frequency by 22 ± 2% from 54 ± 8 to 66 ± 10 Hz (*p*_ANOVA_ = 0.037; *p*_B_ = 0.0005), with no change in amplitude. This experiment suggests that the increase in frequency remained after a depolarization. However, in this case, the increase was smaller than that during control (*p*_t_ < 0.01).

### Interaction With Other IP_3_ Producing Receptors

As a number of different G_q_-linked receptors are likely located on nerve terminals, there is the potential for occlusion between different receptors; *i.e.*, if one receptor is activated, co-activation of another receptor may be occluded. One such class are the group I metabotropic glutamate receptors (mGluR1 and 5), which have been shown to increase presynaptic IP_3_ concentration (Murphy and Miller, 1989).

We tested for occlusion between group I mGluRs and α_1_-ARs by first activating the former with 30 μM DHPG followed by co-application of 10 μM NA. Results of a single experiment are illustrated in **Figures 5A–D**. DHPG significantly increased the mEPSC frequency by 52 ± 9% from 36 ± 2 to 55 ± 1 Hz (*p*_KS_ < 10^-6^), without significantly altering the amplitude. However, when NA was co-applied, no further increase of either mEPSC frequency or amplitude was detected (55 ± 1 Hz; -14.6 ± 0.1 *vs.* -15.4 ± 0.1 pA, respectively; **Figure 5D**). In 9 out of 10 such experiments, application of DHPG increased the mEPSC frequency by 31 ± 5% from 41 ± 3 to 53 ± 4 Hz (*p*_ANOVA_ < 0.001; *p*_B_ < 10^-4^; **Figure 5E**). Subsequent co-application of NA was unable to further increase the mEPSC frequency (48 ± 4 Hz; *p*_B_ = 0.03). This result indicates that occlusion between the two signaling cascades can occur. This may be explained by either a limited concentration of PIP_2_ in the membrane or saturation of the respective receptors on the presynaptic stores.

## Discussion

In this paper we focussed on how NA modulates excitatory miniature release and verified the signaling steps involved. We found that in a subset of pyramidal cells (51%), the addition of ≥1 μM NA or CO to the superfusate increased the mEPSC frequency by 64 ± 7% with no detectable effect on the amplitude. As no changes to the amplitude and time course of the mEPSCs were uncovered, the frequency increase by NA is largely presynaptic. We provide strong evidence that this increase is predominantly caused by activation of α_1_-ARs. We further verified the signaling steps downstream of α_1_-AR activation and found that blocking either PLCβ or IP_3_Rs averted the α_1_-AR-mediated increase in mEPSC frequency. Likewise, chelation of intracellular Ca^2+^ via BAPTA-AM or store depletion had the same effect. These data are consistent with a “classic” α_1_-AR signaling cascade in which PIP_2_ hydrolysis causes DAG and IP_3_ production, the latter of which binds to IP_3_R to cause Ca^2+^ release from stores. We also found that if group I metabotropic glutamate receptors were activated by DHPG, subsequent co-application of NA prevented a further increase in mEPSC frequency.

### Presynaptic Action of NA

As disambiguation of mEPSC data from pre- and postsynaptic contributions is notoriously difficult and control experiments are difficult to perform that only affect the same synaptic population in neocortex from which the mEPSC originate, we were left with gaining confidence about the site of action by excluding a potential postsynaptic contribution using iontophoresis of AMPA onto dendrites using fine electrodes with and without 10 μM NA in the superfusate. Furthermore, these findings were cross-checked for consistency with the average mEPSC amplitudes and time courses.

We did not observe any significant changes in mEPSC or iEPSC amplitude or time course, even though a small drop in *R*_in_ was seen and an increased excitability after NA exposure have been reported in other preparations (Devilbiss and Waterhouse, 2000; Kobayashi et al., 2009; Zhang et al., 2013). Consequently, without any significant postsynaptic contribution to the mEPSC, the frequency increase is largely attributable to the presynapse.

We cannot rule out the possibility that iontophoresis activated only extrasynaptic AMPA receptors. We think that this is unlikely as we kept the iEPSC amplitudes realistically small (∼-50 pA) and because small movements of <1 μm in any direction made the iEPSCs very much smaller, suggesting that a high density of receptors in a confined area generated these currents, something that would not necessarily be the case for extrasynaptic AMPA receptors (Tanaka et al., 2005).

The iontophoretic data also largely rules out the possibility that NA acted via unsilencing of postsynaptic AMPA receptors as the iEPSCs amplitude should have become bigger. As unsilencing is typically linked to a Ca^2+^ rise (Hanse et al., 2013), BAPTA-AM loading should have prevented a frequency increase; however, there was a decrease by 24 ± 6%. All these negative results when testing for postsynaptic effects together with the increase in mEPSC frequency suggest that NA acted on presynaptic nerve terminals.

### NA Activates α_1_-ARs

We mostly used a saturating concentration of 10 μM NA in keeping with other studies (Madison and Nicoll, 1988; Scanziani et al., 1993; Delaney et al., 2007; Zhang et al., 2013). This concentration was smaller than that used by others (Marek and Aghajanian, 1999; Gordon and Bains, 2005), but higher than that applied by Nakamura et al. (2013). We further determined that the *EC*_50_ equalled 0.26 ± 0.19 μM, a value very similar to what others have reported (Mohell et al., 1983; Arias-Montaño et al., 1999).

We found that the mEPSC frequency increase did not significantly involve either α_2_- or β-ARs. This increase is comparable to that observed by Chen et al. (2006), but much smaller than that observed by Marek and Aghajanian (1999). The likely explanation for the latter may be unspecific binding of 100 μM NA to seroton- and/or dopaminergic receptors. We argue that we have provided very strong evidence for α_1_-AR involvement from five lines of evidence. By using, (1) α_1_-AR specific agonists and antagonists, the increase in frequency was mimicked or averted, respectively; (2) blocking ARs other than α_1_ did not alter the frequency increase; (3) the *EC*_50_ value for NA binding at α_1_-AR obtained corresponds very well to that reported for α_1_-AR (Mohell et al., 1983; Arias-Montaño et al., 1999); (4) the displacement of NA at the α_1_-AR by a competitive antagonist abolished the increase; and (5) the downstream signaling after α_1_-AR activation via G_q_ is consistent with the “classic” signaling cascade which causes Ca^2+^ release from stores.

Based on the pharmacological data provided, we are unable to comment on which subtype(s) of the α_1_-AR is/are involved. Our data is compatible with the observation that, using *in situ* hybridization, mRNAs for both α_1A_- and α_1B_-ARs are diffusely expressed in the upper layers of rat cortex (McCune et al., 1993).

### Activation of a Subset of Pyramidal Cells

In this data set, 51% of pyramidal cells were identified as responders based on the significance level reached with the KS statistic for the mEPSC frequency. We stress that even when the frequency increases in responders and non-responders were pooled, a highly significant increase remained; *i.e.*, the increase is robust to the “dilution” by non-responders (*p*_pt_ = 3⋅10^-9^). Because this classification rests on an arbitrary cut-off value, we cannot rule out the possibility that some cells might have been mis-classified, and, if the cut-off was more lenient, that the numbers of responders might be larger. We don’t think that this is necessarily the case as when the frequency changes in all non-responders were averaged, the value was 0 ± 3%, *i.e.*, not different from zero. In addition, the fact that both the lower initial mEPSC frequency and the decrease in *R*_in_ upon NA exposure were significantly different in responders compared to non-responders further strengthens the basis for this classification.

Because most of our recordings were restricted to excitatory pyramidal cells in the upper layer II of this area of cortex, we don’t think that this grouping is due to some cells belonging to a different layer and projecting to different targets. We are not aware such a classification had been used in the context with NA. We note that similar classifications have been applied to pyramidal cells in prefrontal cortex exposed to serotonin (Spain, 1994; Dembrow et al., 2010; Avesar and Gulledge, 2012; Elliott et al., 2018), adenosine (van Aerde et al., 2015) or dopamine (Gee et al., 2012). This study extends the notion of subsets to include NA. Because our experiments were not designed to identify the factors that give rise to these groupings, we are unable to comment on the specificity and sensitivity of this classification.

### Mechanism of mEPSC Frequency Increase

Because of the possibility that different signaling cascades may be involved downstream of receptor activation and because these mechanisms have been poorly explored in somatosensory cortex, we carefully verified each step in the signaling cascade.

To inhibit PLCβ, we used the phospholipase C inhibitor edelfosine (ES). ES produces a much cleaner inhibition of PLCβ than the alternative U73122 (Horowitz et al., 2005). We found that when PLCβ was blocked by ES, the mEPSC frequency decreased by 22 ± 3%. This decrease could be due to two explanations: firstly, due to tonic receptor activation, and secondly, due to constitutive PLCβ activity, independent of receptor activation. The latter explanation seems most likely because after tissue slicing, action potential mediated NA release from relevant nerve terminals most likely ceases. This idea is consistent with a report from *Drosophila* photoreceptors, where there is constitutive activity of PLCβ (Hardie et al., 2004).

When IP_3_Rs were blocked by 16 μM 2-APB, a 34 ± 5% decrease in mEPSC frequency was seen. This experiment shows that, first, the extent of the frequency reduction is not different to that when PLCβ is blocked (Simkus and Stricker, 2002b), and second, 2-APB likely blocks IP_3_Rs. We cannot rule out the possibility that 2-APB was affecting another target like a TRP channel. However, we note that in a previous study (Simkus and Stricker, 2002a), lowering extracellular Ca^2+^ or blocking channels with divalent ions did not lower the mEPSC frequency, rendering the involvement of a TRP channel unlikely. The outcome of this experiment is also inconsistent with the idea that the main signaling molecule is DAG, which activates PKC as reported by Kobayashi et al. (2008). In this scenario, as 2-APB blocks IP_3_R but does not interfere with the DAG arm of the cascade, an increase in mEPSC frequency after exposure to NA should have been observed.

Consistent with our earlier report, we did not observe any changes in mEPSC frequency with CPA alone, but required a prolonged depolarization to force store depletion (Simkus and Stricker, 2002b). We do not think that this depolarization impacted on vesicle release unrelated to Ca^2+^ stores for the following reasons. (1) The recovery rate of a vesicle is about 1 s as we (Fuhrmann et al., 2004) and others (for example Dittman and Regehr, 1998) have determined. Because the membrane potential returned to resting conditions over many minutes, this time is likely sufficient to replenish most of the vesicle pools. (2) We provided evidence in a set of 19 experiments that the K^+^ depolarization neither affected the mEPSC frequency nor amplitude. (3) In addition, we show that the decrease in mEPSC frequency was not different to when intracellular Ca^2+^ was chelated with BAPTA-AM (*p*_O_ = 0.18), given that voltage-dependent Ca^2+^ channels are not involved (Simkus and Stricker, 2002b). If the depolarization did something different to the release machinery, the two numbers would unlikely have been indistinguishable. (4) There is evidence in the literature that nerve terminals recover functionally well after a short K^+^ depolarization (for example Sitges and Galindo, 2005). These different lines of evidence, reasons and the internal consistency thereof, make us confident that release from presynaptic Ca^2+^ stores drives the NA-mediated increase in mEPSC frequency. Consequently, we are convinced that a “classic” signaling cascade downstream of α_1_-AR activation is in place.

### Occlusion When Another G_q_-Cascade Is Co-activated

We observed that when group I mGluRs were activated by DHPG (Abe et al., 1992), co-exposure with NA occluded a further increase in mEPSC frequency. We have three potential explanations for this observation. Firstly, IP_3_ production could be limited due to restricted availability of PIP_2_ in the membrane. Indeed, Falkenburger et al. (2010) found direct evidence for this idea by reporting that the time constant of replenishment of PIP_2_ in the membrane is about 130 s, whereas the hydrolysis of PIP_2_ is much faster. Secondly, if the number of IP_3_ receptors on stores was small and the IP_3_ concentration saturating, a further increase in the concentration of IP_3_ would not result in additional Ca^2+^ store release. Thirdly, the stores may deplete quickly of Ca^2+^. The latter is unlikely as we have reported that the application of 10 mM caffeine for more than 20 min was insufficient to significantly deplete Ca^2+^ stores (Simkus and Stricker, 2002b).

We note that we used saturating concentrations of both DHPG and NA. However, if non-saturating concentrations were used, much subtler interactions may be detectable, or the observations may depend on the order in which the different receptors were activated.

### Implications

The fact that NA increases mEPSC frequency in a subset of cells raises the possibility that the action of NA may be restricted to specific cells within particular networks. In these cells, by increasing the frequency of mEPSCs in the neocortical tissue, NA could have an important role as a neurotrophic factor in postnatal dendritic spine development as the spontaneous release of glutamate was found to be essential for the maintenance of dendritic spines (McKinney et al., 1999). This idea is in agreement with the observation that NA shapes dendrite elongation and spine formation in the neocortex of rats (Felten et al., 1982). At the functional level, the impact of an increased mEPSC frequency remains unclear, but changes either to the signal-to-noise ratio when background noise is elevated (Servan-Schreiber et al., 1990; Hasselmo et al., 1997), or to synaptic integration due to the observed reduction in *R*_in_ (Foehring et al., 1989), to the extent of homeostatic plasticity (Turrigiano et al., 1998) or to long-term plasticity (Izumi, 1992; Katsuki et al., 1997; Kirkwood et al., 1999) may be expected.

Given that activation of presynaptic α_1_-ARs increases the rate of mEPSCs via Ca^2+^ release from presynaptic stores, we would expect that the Ca^2+^ store release had an impact on Ca^2+^ homeostasis within the nerve terminal and may thereby increase the probability of transmitter release and thus increase the action potential mediated EPSC amplitude.

## Ethics Statement

All animal housing, breeding and surgical procedures were approved by the Animal Experimentation Ethics Committee of the Australian National University and conform to the guidelines of the National Health and Medical Research Council (NHMRC) of Australia.

## Author Contributions

JC, LL, and FA performed the experiments and analyzed the data. JC, LL, FA, and CS interpreted the results of experiments, prepared figures, and drafted the manuscript. CS conceived and designed the research.

## Conflict of Interest Statement

The authors declare that the research was conducted in the absence of any commercial or financial relationships that could be construed as a potential conflict of interest. The reviewer CG and handling Editor declared their shared affiliation.
